# Rootstock Influence on Wine Aroma Compounds and Sensory Perception: A Review

**DOI:** 10.3390/foods14213593

**Published:** 2025-10-22

**Authors:** Laura Farris, Elisa Marguerit, Georgia Lytra, Jean-Christophe Barbe

**Affiliations:** 1 Univ. Bordeaux, INRAE, Bordeaux INP, Bordeaux Sciences Agro, UMR 1366, OENO, ISVV, F-33140 Villenave d’Ornon, France; laura.farris@agro-bordeaux.fr (L.F.); georgia.lytra@agro-bordeaux.fr (G.L.); 2 EGFV, Univ. Bordeaux, Bordeaux Sciences Agro, INRAE, ISVV, F-33882 Villenave d’Ornon, France; elisa.marguerit@agro-bordeaux.fr

**Keywords:** grapevine, rootstock, wine aroma, chemical composition, sensory impact

## Abstract

Rootstocks play a crucial role in viticulture because of both their agronomic benefits and impact on grape and wine chemical composition. Their influence on vigor, yield, and phenolic maturity has been widely studied, but only recently have studies begun to examine their effects on wine aroma compounds and sensory profiles. This review compiles findings of studies investigating 33 rootstocks for their influence on wine aroma composition and 29 rootstocks for sensory evaluation across 10 red and four white grape varieties. The most studied rootstocks include 1103 Paulsen, 110 Richter, Sélection Oppenheim 4 and 101-14 Millardet et de Grasset. Shiraz, Merlot, and Cabernet-Sauvignon are the most frequently assessed varieties. Despite growing works in this research area, results are highly variable due to differences in experimental design, winemaking protocols, and sensory methodologies. Only five studies have simultaneously analyzed both wine aroma compounds and sensory attributes. While some consistent trends emerge for certain aroma compounds, such as β-damascenone or linalool, most findings remain context-dependent. This review emphasizes a lack of standardization, including that of winemaking conditions, and highlights the critical need for multi-site, multi-year studies to better understand rootstock effects on wine aroma and sensory quality.

## 1. Introduction

The aroma profile of wine is a central determinant of its typicity [1], perceived quality [2], and consumer acceptance [3]. It results from complex interactions between pedoclimatic conditions [4], agronomic practices [5], and the winemaking process [6], as well as the plant material used, such as the grape variety [7] and rootstock [8].

Initially developed for their resistance to phylloxera, a soil-borne aphid, rootstocks are fundamental in modern viticulture, providing benefits such as influencing plant vigor and yield, and enhancing essential adaptability to environmental conditions, especially in the context of global warming characterized by rising temperatures, increased frequency of drought, and extreme weather events [9].

As demonstrated by numerous studies, rootstocks significantly influence the chemical composition of the grapes, particularly the levels of sugar and organic acids [10,11], which impact the potential alcohol content and perceived acidity of the wine. Furthermore, they affect the phenolic composition of the grape [12,13], influencing the wine’s color, structure, and aging potential. Among these influences, their effect on wine aroma compounds and sensory perception has become a subject of increasing interest and constitutes the core of this review (Table 1).

This research on wine aroma compounds has involved a diverse range of grape varieties, encompassing a total of 33 distinct rootstocks. The most frequently analyzed rootstocks include 110 Richter (*n* = 8), 1103 Paulsen (*n* = 8), 3309 Couderc (*n* = 7), Sélection Oppenheim 4 (*n* = 7), 101-14 Millardet et de Grasset (*n* = 5), and Gravesac (*n* = 4); own-rooted vine were also assessed (*n* = 6). These studies included 10 red and two white grape varieties, Shiraz and Merlot being the most frequently studied.

Complementing these analyses, numerous studies have also assessed the effect of rootstocks on the sensory perception of wine. A comprehensive review of the existing literature reveals that 29 rootstocks have been the focus of prior studies. The most frequently studied rootstocks for sensory evaluation are 1103 Paulsen (*n* = 9), 110 Richter (*n* = 7), 101-14 Millardet et de Grasset (*n* = 6), 140 Ruggeri, Dog Ridge (*n* = 4), and 3309 Couderc, Gravesac, Sélection Oppenheim 4, Fercal and Ramsey (*n* = 3). Six red and two white grape varieties have been analyzed in these studies, involving a variety of environmental conditions and viticultural practices. Shiraz and Cabernet-Sauvignon emerged as the most frequently studied grape varieties in these sensory assessments. Notably, only five studies exclusively on red grape varieties have simultaneously evaluated the effect of rootstocks on both aroma compounds and sensory perception.

Despite growing interest in this research area, drawing consistent and comprehensive conclusions from existing literature on rootstock effects on wine aroma compounds and their sensory perception is challenging due to a limited number of dedicated studies and the heterogeneity of methodologies used.

This review aims to synthesize current findings by highlighting general trends and identifying major gaps in the literature. A comprehensive understanding of the influence of rootstocks on aroma is crucial for optimizing viticultural practices and developing adaptation strategies to adapt to current viticultural challenges.

To facilitate the understanding of rootstock impacts on wine aroma composition, the subsequent sections summarize the findings from the literature in tables organized by aroma compound family. Each table detail the aroma compound, its concentration range observed in both red and white wines (across all grape varieties), and the statistically significant rootstock effect observed: in all cases (Yes), in no cases (No), or only in certain cases (Sometimes), depending on the vintage. Particular attention was also given to whether reported concentrations could have a sensory impact, whether differences between rootstocks seemed meaningful, and whether recurring trends associated with specific rootstocks could be identified.

**Table 1:** Summary of studies involving rootstocks with focus on wine aroma compounds and sensory profile.

Grape Variety	Rootstock (*n*)	Rootstocks (List)	Vintage (*n*)	Aroma Families	Sensory Methods
*Albarín Negro* [14]	5	101-14MGt, 110R, 196-17Cl, 3309C, Rupestris du Lot	3	Esters, Alcohols	OIV scoring sheet (visual, odor, taste, global evaluation)
*Alicante Bouschet* [15]	2	1103P, IAC572	2 harvest time		QDA (16 attributes), 10-point US scale, 12 experienced wine tasters
*Cabernet-Sauvignon* [16]	15	101-14MGt, 110R, 1103P, 161-49C, 3309C, 420A, 5BB, 99R, Dog Ridge, Fercal, Gravesac, Rupestris du Lot, SO4, Solferino, Isabel	3	Esters, Alcohols	
*Cabernet-Sauvignon* [17]	15	101-14MGt, 110R, 1103P, 161-49C, 3309C, 420A, 5BB, 99R, Dog Ridge, Fercal, Gravesac, Rupestris du Lot, SO4, Solferino, Isabel	1		DA (22 attributes), 9 cm US scale, 12 panelists with sensory analysis background
*Cabernet-Sauvignon* [18]	8	Dog Ridge, 110R, 140Ru, 1103P, 101-14MGt, SO4, Fercal, Gravesac	1		DA (9 attributes), 7-point US scale, 12 trained panelists
*Chambourcin* [19]	4	1103P, 3309C, Own roots, SO4	2	Monoterpenic compounds, Norisoprenoids, Esters,	
*Marselan* [20]	3	1103P, 5BB, Own roots	1	Monoterpenic compounds, Esters, C6 Compounds, Alcohols	
*Merlot* [20]	4	1103P, 5BB, Own roots, SO4	1	Monoterpenic compounds, Esters, C6 compounds, Alcohols	
*Merlot* [21]	9	101-14MGt, 1103P, 110R, 140Ru, 4453M, 99R, Gravesac, Own roots, SO4	1	Esters, C6 compounds, Alcohols	
*Monastrell* [22]	5	1103P, 110R, 140Ru, 161-49C, 41B	2	Monoterpenic compounds, Esters, C6 compounds, Alcohols	DT using INDO scoring sheet (structured visual, olfactory, gustatory phases + harmony), 5 trained expert tasters, positive/negative attribute scoring system
*Pinot noir* [23]	15	101-14Mgt, 110R, 1103P, 3309C, 5C, C113, C114, C20, C29, M5489, M5512, M6262, Own roots, SO4, Schwarzmann	2	Monoterpenic compounds, Esters, Alcohols	
*Shiraz* [8]	4	1103P, 110R, Own roots, Schwarzmann	2	Monoterpenic compounds, Norisoprenoids, Esters, C6 compounds, Alcohols	DA (25 attributes), 15 cm US scale, 15 and 12 trained panelists (2010, 2011); Wine quality: 20-pt Wine Show score, 8 and 12 experts (2010, 2011)
*Shiraz* [24]	3	Dog Ridge, M6262, Ramsey	1	MPs, Monoterpenic and C6 compounds	Informal evaluation, free-choice descriptive notes, 8 trained judges
*Shiraz* [25]	8	101-14MGt, 110R, 1103P, 140Ru, M5489, M5512, M6262, Ramsey	2		DA, 15 cm US scale, 11 and 12 trained panelists (2012 and 2013), training with reference standards
*Shiraz* [26]	2	5C, Gravesac	1		DA (13 attributes), 15 cm US scale, 10 trained panelists with previous wine evaluation experience
*Shiraz* [27]	2	1103P, IAC313	3 + 4 seasons		QDA (16 attributes), 10-point US scale, 12 experienced wine tasters
*Verdejo Negro* [28]	3	101-14MGt, 196-17Cl, 3309C	3	Esters, Alcohols	OIV scoring sheet (visual, nose, taste, global evaluation), median scores, 6 experienced tasters
*Albariño* [29]	9	110R, 161-46C, 196-17Cl, 3309C, 41B, 420A, Gravesac, RGM, SO4	2	Monoterpenic compounds, Norisoprenoids, Esters, C6 compounds	
*Riesling* [30]	6	110R, 140Ru, 3309C, Börner, Gravesac, SO4	2	Monoterpenic compounds, Norisoprenoids, Esters, C6 compounds	
*Sauvignon blanc* [31]	7	1103P, 110R, 140Ru, Dog Ridge, Fercal, Ramsey (Salt Creek), SO4	1		Hedonic test, 9-point scale (color, appearance, flavor, taste, overall acceptability), 6 assessors
*Vermentino* [32]	4	101-14MGt, 1103P, Harmony, VR043-43	3		Quantitative evaluation, 0–10 intensity scale (visual, olfactory, taste, overall balance), 10 trained judges

For each study, the grape variety, number and list of rootstocks (in alphabetical order), number of vintages or seasons, authors, main aroma compound families analyzed, and sensory evaluation method are provided. The following abbreviations are used to summarize the sensory evaluation protocols: DA (Descriptive Analysis), QDA (Quantitative Descriptive Analysis), DT (Difference Testing), US scale (Unstructured scale), OIV (Official sensory sheet from the Organisation Internationale de la Vigne et du Vin). Rootstock abbreviations used in the table are as follows: 101-14MGt—101-14 Millardet et de Grasset; 110R—110 Richter; 1103P—1103 Paulsen; 140Ru—140 Ruggeri; 161-49C—161-49 Couderc; 196-17Cl—196-17 Castel; 3309C—3309 Couderc; 41B—41 B Millardet et de Grasset; 420A—420 A Millardet et de Grasset; 4453M—4453 Malègue; 5BB—Kober 5 BB; 5C—Teleki 5 C; C113, C114, C20, C29—CSIRO selections of C rootstocks; M5489, M5512, M6262—Merbein selections; RGM—Riparia Gloire de Montpellier; SO4—Sélection Oppenheim 4.

## 2. Impact of Rootstock on Aroma Compounds

Rootstock has been reported to affect a wide range of chemical families that contribute to wine aroma, including methoxypyrazines, monoterpenic compounds, C13-norisoprenoids, esters, and alcohols (Table 2, Table 3 and Table 4). The families are presented in a consistent order, with grape-derived compounds listed first, followed by fermentation-derived compounds. In the following sections, each family is examined in turn, with emphasis on key representative compounds. Aroma compounds that have been investigated only occasionally or in single studies are not included in this work.

### 2.1. 2-Methoxy-3-Isobutylpyrazine

2-methoxy-3-isobutylpyrazine (IBMP) is a nitrogen heterocycle derived from the metabolism of amino acids, which are naturally occurring aromatic compounds found in plants [33]. IBMP is responsible for the characteristic green bell pepper aroma in wine.

The aromatic properties of these odor-active compounds in wine have been extensively documented [34,35]. IBMP is the most common methoxypyrazine (MP) found in grapes. It is primarily located in the stems, but it is also present in the berry skin in significantly higher concentrations than in the seeds and pulp [36]. Characterized by a very low perception threshold (PT) of 15 ng/L in red wines, IBMP is well-known for its role in vegetal aromas, with high levels typically associated with under-ripe grapes and lower quality wines [36].

The impact of rootstock on MPs concentration has been recently explored on Cabernet-Sauvignon grapes berries [37] and Shiraz rachis [38]. In Cabernet-Sauvignon, a significant difference in IBMP concentration was observed over two vintages, with higher concentrations found in berries from vines grafted onto 140Ru (15.8 ng/kg) and 1103P (9.24 ng/kg) rootstocks. Conversely, Börner was associated with the lowest concentrations (1.97 ng/kg) [37]. These IBMP concentration differences identified in grape berries by Sanders et al. [37] could potentially have a significant impact even after vinification. Indeed, since IBMP is mainly present under its free form in grapes and transferable to wine in red winemaking conditions, such observed differences may have an impact on wine aroma, although this remains to be confirmed. Furthermore, only one recent study by Capone et al. [24] investigated MPs in Shiraz wines produced from three rootstocks. The available data, based on a single measurement per wine, limit the robustness of the conclusions. This study found that IBMP was the only MP detected in the wines, with higher concentrations in wine from Dog Ridge (8 ng/L) and Ramsey (7 ng/L) compared to that from Merbein 6262 (≤0.5 ng/L). Although the IBMP concentrations found were below the PT of 15 ng/L and based on a limited number of measurements, these results still highlighted the concentration differences in wines from different rootstocks. This observation is particularly interesting for grape varieties inherently characterized by their potential to produce elevated IBMP levels, such as Cabernet-Sauvignon and Carménère.

Overall, while rootstock influence on this specific methoxypyrazine is evident in berries and rachis, definitive conclusions for wine quality remain elusive. Interestingly, Sanders et al. [37] also reported a correlation between vine vigor and IBMP concentration in Cabernet-Sauvignon berries, suggesting that rootstocks promoting higher vigor could indirectly increase vegetal notes in wines. As vigor is closely linked to nitrogen status, a rootstock-mediated effect on nitrogen uptake may likewise contribute to IBMP variation. Nevertheless, such relationships have not yet been demonstrated in wines, highlighting the need for more integrated studies.

### 2.2. Monoterpenic Compounds

Monoterpenic compounds are composed of 10-carbon atoms, formed from two five-carbon isoprene units [39,40]. Approximately 50 monoterpenic compounds are currently known, among which linalool, geraniol, citronellol, and α-terpineol are the most abundant in Muscat grape varieties, where they are responsible for the specific aroma [41]. These compounds are characterized by floral descriptors. Their PT in model wine solutions are 25, 30, 100, and 250 µg/L, respectively [42].

Several studies have explored the effect of rootstock on monoterpenic compounds, although reported results are often inconsistent across experiments, as summarized in Table 2. The observed effects vary significantly depending on the specific cultivar and rootstock combination, making it challenging to draw definitive conclusions. While red grape varieties are not generally associated with high levels of monoterpenes, white cultivars such as Riesling [43] and Albariño [44] typically exhibit higher concentrations, which may contribute to muscat-like aromatic notes. Nevertheless, a rootstock effect has been highlighted on linalool, geraniol, α-terpineol, citronellol, and hotrienol, although the consistency of these effects differs across studies, as summarized in Table 2. This variability may partly reflect vintage conditions and their interaction with rootstock-induced differences in viticultural parameters, such as vigor or yield, which can in turn affect berry ripening. However, these factors have rarely been studied together in the available literature.

Although findings across studies are mixed, linalool often exhibits rootstock effects, with some indicating partial or no significant impact, as summarized in Table 2. A comprehensive study on Riesling using six rootstocks showed a consistent influence of both rootstock and vintage on linalool levels across two years [30]: linalool concentrations in wines ranged from 54 to 75 µg/L in one vintage and 68 to 127 µg/L in the other. However, the relative contribution of vintage versus rootstock was not clearly quantified, and climatic conditions were only reported on an annual scale, rather than over the ripening period, which limits the interpretation of these results.

These levels were generally higher than those typically reported in the literature for Riesling (ranging from nd to 230 µg/L, with a median of 12 µg/L based on 53 studies; [42]). Furthermore, the concentrations observed in this study consistently exceeded the PT of 50 µg/L reported in wine [45], suggesting a potential sensory contribution of linalool to the wine’s aroma. This was corroborated by Garbay et al. [46], who demonstrated that the addition of 38 µg/L of linalool to a fruity aromatic reconstitution significantly enhances fresh blueberry and jammy blueberry notes.

Regarding rootstock trends, Ziegler et al. [30] highlighted a consistent pattern: wine from Börner and 140Ru showed the lowest levels of linalool, while wine from 3309C, 110R and SO4 exhibited the highest concentrations across both vintages of the study. Similarly, Vilanova et al. [29] observed a rootstock (*n* = 9) effect on linalool concentrations in Albariño wine, though this was only evident in one of the two vintages studied. Concentrations ranged from 32 to 71 µg/L. While these concentrations were lower compared to those reported for Riesling by Ziegler et al. [30], a similar rootstock trend to Riesling was observed in Albariño wine, with wine from 110R and 3309C being associated with higher levels in that particular vintage. By contrast, for the same vintage, wine from SO4 showed lower concentrations, highlighting the complexity of cultivar-rootstock interactions and the importance of environmental factors.

**Table 2:** Effect of rootstock on concentration range of monoterpenic and C13-norisoprenoids compounds in wine.

Aroma Compound	Concentration Range in Wine (µg/L)		Grape Variety Studied		Rootstock Effect		
	White	Red	White	Red	Yes	No	Sometimes
**Monoterpenic compounds**							
Linalool	32–127 [29,30]	1.6–15 [19,20]	Riesling, Albariño	Merlot, Marselan, Monastrell, Chambourcin	[19,20,30]	[20,22]	[29]
Geraniol		1.8–32 [20]		Merlot, Marselan, Shiraz, Pinot noir	[20]	[23]	[8]
α–terpineol	6–11 [29]	0.86–1.05 [20]	Albariño	Merlot, Marselan, Monastrell	[20]	[22]	[29]
Citronellol				Merlot, Marselan, Monastrell, Shiraz		[20,22]	[8]
hotrienol	8–43 [29]		Albariño				[29]
**C13-Norisoprenoids**							
β-damascenone	0.6–14 [29,30]	4.6–13.3 [19]	Riesling, Albariño	Chambourcin, Shiraz	[19,30]		[8,29]
1,1,6-trimethyl-1,2-dihydronaphthalene	2.3–12 [30]	0.4–0.6 [19]	Riesling	Chambourcin	[30]		[19]
Vitispiranes	2.7–10.1 [30]		Riesling		[30]		

Concentration range: mean concentration recorded for rootstocks associated with the lowest and highest concentration only when a statistically significant rootstock effect was observed. Rootstock effect: the categories indicate whether a statistically significant effect of the rootstock was observed in all cases (Yes), in no cases (No), or only in certain cases, varying depending on the vintage (Sometimes).

In red wine, Awale et al. [19] reported consistent significant rootstock effects on linalool in Chambourcin wine. Linalool concentrations ranged from 8.4 to 10 µg/L in one vintage and 6.9 to 15 µg/L in the other. Wine from own-rooted vines consistently exhibited lower linalool levels, while 1103P was associated with the highest concentrations. However, it is important to note that these levels remain well below the PT reported for model wine solutions [42], which limits the potential impact of this compound on the overall wine profile.

As well as linalool, other monoterpenic compounds also exhibited variable responses to rootstock effect, as shown in Table 2. For geraniol, a rootstock effect was observed only in red wine. Li et al. [20] reported this effect in Marselan and Merlot wines, but the highest concentration recorded over two years (32 µg/L) remains well below the PT of 130 µg/L reported in wine [45]. Although the impact of monoterpenic compounds in mixtures has long been highlighted [47], the concentrations observed here are probably too low to exert any perceptible influence on aroma. In line with these findings, a partial rootstock effect on geraniol was noted by Martilla et al. [8], who compared own-rooted Shiraz vines with three rootstocks over two vintages. In a semi quantitative approach, they showed that wine from 1103P and own-rooted vines exhibited significantly higher concentrations compared to 110R and Schwarzmann rootstocks. However, given the variability and the limited consistency across studies, drawing definitive conclusions from these compounds remains challenging, and it is not possible to identify similar rootstock trends for geraniol across these studies.

A similar conclusion can be made for citronellol, α-terpineol, and hotrienol. Although rootstock effects have been highlighted in some studies (Table 2), the recorded concentrations of citronellol and α-terpineol are well below their respective PT in model wine solutions: 100 and 250 µg/L, respectively [42]. Hotrienol with a PT of 110 µg/L in wine [48], has only been studied in the white wine of Albariño, where a rootstock effect was observed in only one vintage. The concentration range was found to be quite broad, from 8 to 43 µg/L, with wine from SO4 being associated with the lowest concentrations and 3309C with the highest [29].

To sum up, while the influence of rootstock on monoterpenic compounds is generally characterized by limited and inconsistent findings, linalool nonetheless stands out. It frequently exhibits clearer rootstock-related trends, with two specific rootstocks, 3309C and 110R, showing consistent associations with higher levels across cultivars and studies. These repeated observations suggest that these rootstocks may consistently influence linalool biosynthesis and its concentration in the resulting wine. For other monoterpenic compounds like geraniol, citronellol, or α-terpineol, no consistent rootstock-related trends have been identified across studies, and the available data is considered insufficient to establish robust generalizations or consistent trends.

### 2.3. C13-Norisoprenoids

C13-norisoprenoids, characterized by a cyclic structure of 13 carbon atoms, are derived from carotenoids. They are the predominant norisoprenoids in grapes and are able to play an important role due to their aromatic properties in wine [49]. The main C13-norisoprenoids found in wine include β-damascenone (with fruity, floral, and baked apple notes), β-ionone (violet, raspberry, and rose aromas), 1,1,6-trimethyl-1,2-dihydronaphthalene (TDN; with kerosene and petrol characteristics), and vitispiranes (eucalyptus, camphor, and vegetable nuances) [42]. Their PTs are 0.05 and 0.09 µg/L for β-damascenone and β-ionone in model wine solutions, and 20 and 800 µg/L for TDN and vitispiranes in white wine, respectively [42].

The influence of rootstock on C13-norisoprenoids has been investigated in both white and red wines (Table 2). Impact varies widely depending on specific compound, grape variety and environmental conditions. Some studies report consistent effects across multiple vintages, while others have found a significant rootstock effect in only one year. Among these compounds, β-damascenone is the most studied and has shown consistent rootstock effects across multiple vintages in Riesling and Chambourcin. Similarly, in Albariño and Shiraz, a rootstock effect was observed in only one of the two years studied (Table 2). However, the role of vintage conditions has not been consistently documented across studies, making it difficult to disentangle rootstock effects from year-to-year climatic variability. Similarly, a stable rootstock effect has been observed for TDN in Riesling across vintages, but in Chambourcin the effect was only significant in one year. Vitispiranes were also influenced by rootstock across multiple vintages in Riesling (Table 2).

In Riesling, the observed ranges of β-damascenone concentrations were similar across the two vintages studied, from 0.6 to 2.6 µg/L and 1.1–2.5 µg/L [30], aligning with concentrations reported in the literature [42]. In Albariño, overall concentrations were even higher, ranging from 6 to 19 µg/L [29], which is in agreement with other reported studies for Albariño cultivated in different Spanish regions (around 20 μg/L; [50]. These concentrations are remarkably high, both in comparison to typical levels found in wines and considering β-damascenone’s extremely low PT.

Regarding rootstock trends in Riesling, wine from Börner (2.5–2.1 µg/L), SO4 (2.5–1.7 µg/L), and 3309C (2.2–2.6 µg/L) were consistently associated with the highest concentrations of β-damascenone across both vintages, while wines from 140Ru (1.1–1 µg/L), 110R (1.5–0.8 µg/L), and Gravesac (1.6–0.6 µg/L) were associated with the lowest [30]. Regarding Albariño [29], common rootstocks like 110R and Gravesac (both 14 µg/L in Albariño) showed an opposite trend to Riesling in one vintage, being associated with higher concentrations of β-damascenone. By contrast, 3309C displayed the same trend, consistently leading to the highest concentrations in both Riesling and Albariño wines.

Similarly, in the hybrid cultivar Chambourcin rootstock effects on β-damascenone concentrations were observed, with rootstocks being associated with the highest concentrations consistently across both vintages. Wines from own-rooted vines (5.7 and 13.3 µg/L) and 3309C (4.6 and 13 µg/L) displayed the highest average concentration, while wine from 1103P (5 and 12 µg/L) and SO4 (4.7 and 12 µg/L) were associated with the lowest concentrations in different vintages [19].

In several studies across different cultivars such as Albariño and Chambourcin, β-damascenone concentrations exceeded the (overestimated) PT of 7 µg/L in red wine [51], indicating a possible direct impact on wine aroma. In addition, β-damascenone is known to contribute indirectly through synergistic effects, enhancing fruity notes in model solutions, even at very low concentrations (in the order of 50 ng/L, [51]; 0.85 µg/L, [52]; 1.4 µg/L; [53]). Thus, the analytical differences observed between rootstocks described here could also lead to sensory differences.

Notably, rootstock 3309C has consistently been shown to be associated with higher concentrations of β-damascenone across multiple cultivars, including Chambourcin [19], Albariño [29], and Riesling [30]. This suggests a potentially generalizable tendency for 3309C to enhance β-damascenone levels. By contrast, wines from SO4 were associated with the lowest β-damascenone concentrations in Chambourcin [19], which differs from the results observed in Riesling [30]. Conversely, wine from own-rooted vines were associated with the lowest β-damascenone levels in Shiraz [8], contrasting with those in Chambourcin [19]. Similarly, in wine from 110R, lower levels were observed in Shiraz by Martilla et al. [8], which contrasts with Albariño results [29] but aligns with findings in Riesling [30]. These opposing trends across cultivars highlight the complex and context-dependent nature of rootstock effects on C13-norisoprenoids.

TDN and vitispiranes were significantly influenced by rootstock in both white and red wines (Table 2). In Riesling, TDN concentrations ranged from 5.4 to 12 µg/L and from 2.3 to 10.2 µg/L across two different vintages. Wine from Börner was consistently associated with the highest concentrations, and wines from 3309C and 140Ru with the lowest across these vintages [30]. However, given that the PT for TDN in white wine has been reported to be 20 µg/L [42], concentrations found in Riesling remain well below this threshold, suggesting that they may not have a perceptible impact on the wine’s aroma. Similarly, in Chambourcin wines, concentrations were even lower (Table 2).

Finally, regarding vitispiranes, although a rootstock effect has been highlighted in Riesling, the concentrations observed are well below their reported PT of 800 µg/L in white wine [42]. Concentrations ranged from 2.7 to 10.1 µg/L (SO4-Gravesac) and 3.4 to 8.3 µg/L (140Ru–Börner), with no specific rootstock trends identified [30].

In summary, rootstock exerts a variable yet often significant influence on C13-norisoprenoid concentrations in wine. With its extremely low PT, β-damascenone consistently shows rootstock effects, and some patterns, like the enhancing effect of 3309C, appear to be generalizable across cultivars. However, rootstock effects are highly influenced by vintage and environmental conditions, meaning that contrasting trends can be observed for other compounds or combinations. While compounds like TDN and vitispiranes are influenced by these factors, their concentrations often remain below sensory thresholds.

### 2.4. Esters

Esters contribute decisively to the fruity profile of wines, particularly red wines, with olfactory thresholds ranging from a few to several hundred µg/L depending on the wine structure and matrix [51,52,54,55,56,57]. Their sensory impact depends on concentration, as well as on synergistic or modulatory interactions [46,55,58,59,60]. Most esters are produced by yeast metabolism during alcoholic fermentation, or later mainly through chemical esterification with the corresponding acid. In wines, they are grouped as six subfamilies: fatty acid ethyl esters (FAEEs, including short- and long-chain ethyl esters, C4–C16), alkylated ethyl esters (AEEs, substituted ethyl esters with alkyl group), hydroxylated ethyl esters (HEEs), and alkyl and hydroxyl substituted ethyl esters (AHEEs). Similarly, higher alcohol acetates are subdivided into non-substituted higher alcohol acetates (LHAAs) and substituted higher alcohol acetates (SHAAs). Rootstock effects have been studied on 59 esters; Table 3 presents a selection of these based on quantitative data availability and aromatic relevance. In these studies, grape maturity was generally controlled, and harvests were conducted at similar ripening stages.

#### 2.4.1. Fatty Acid and Alkylated Ethyl Esters

Rootstock effects on FAEE and AEE are variable but recurrent in several key compounds: ethyl butanoate, ethyl hexanoate, ethyl octanoate, ethyl decanoate, and, to a lesser extent, longer-chain esters, such as ethyl dodecanoate, ethyl tetradecenoate, and ethyl hexadecanoate.

**Table 3:** Effect of rootstock on concentration range of esters in wine.

Aroma Compound	Concentration Range in Wine (µg/L)		Grape Variety Studied		Rootstock Effect		
	White	Red	White	Red	Yes	No	Sometimes
**Fatty acid ethyl esters**							
Ethyl butanoate	57–318 [29,30]		Riesling, Albariño	Merlot, Monastrell, Pinot noir	[30]	[21,22,23]	[29]
Ethyl hexanoate	272–1990 [29,30]		Riesling, Albariño	Merlot, Monastrell, Pinot noir, Shiraz	[30]	[21,22,23]	[8,29]
Ethyl octanoate	101–3903 [29,30]	30–1010 [19,21]	Riesling, Albariño	Chambourcin, Merlot, Pinot noir, Shiraz, Monastrell	[8,19,21,30]	[22,23]	[29]
Ethyl decanoate	182–254 [29]	14–260 [19,21]	Albariño	Chambourcin, Pinot noir, Shiraz, Monastrell, Merlot	[21]	[22,23]	[8,19,29]
Ethyl dodecanoate		136–399 [22]		Monastrell, Pinot noir	[22]	[23]	
Ethyl tetradecenoate		3.3–4.6 [19]		Chambourcin			[19]
Ethyl hexadecanoate		34.2–48.8 [19]		Monastrell, Chambourcin		[22]	[19]
**Hydroxylated ethyl esters**							
Ethyl-3-hydroxybutaote		133–187 [22]		Monastrell	[22]		
**Higher alcohol acetates**							
Hexyl acetate	16–171 [29,30]		Riesling, Albariño	Shiraz, Monastrell, Pinot noir	[8,30]	[22,23]	[29]
2-Phenylethyl acetate	18–460 [29,30]	10–46 [19]	Riesling, Albariño	Shiraz, Chambourcin, Pinot noir, Albarín Negro, Verdejo Negro	[8,19,29,30]	[14,23,28]	
2-methylbutyl-acetate	69–104 [30]		Riesling		[30]		
3-methylbutyl-acetate	868–5372 [29,30]	340–1160 [19,21]	Riesling, Albariño	Merlot, Monastrell, Pinot noir, Shiraz, Chambourcin	[21,29,30]	[22,23]	[8,19]

Concentration range: mean concentration recorded for the rootstocks associated with the lowest and highest concentration, only when a statistically significant rootstock effect was observed. Rootstock effect: the categories indicate whether a statistically significant effect of the rootstock was observed in all cases (Yes), in no cases (No), or only in certain cases, varying depending on the vintage (Sometimes).

Ethyl butanoate is consistently influenced by rootstock in white wine. In Riesling, concentrations have been found to range from 209 to 298 µg/L and from 250 to 318 µg/L across two vintages [30]; a consistent rootstock trend was observed in both years, with wines from 3309C and 140Ru containing the highest levels and those from Börner the lowest. Similarly, in Albariño, ethyl butanoate concentrations ranged from 57 to 175 µg/L (161-49C and 110R) in one vintage [29]. However, no consistent rootstock trend was found when comparing the Riesling and Albariño studies. The concentrations observed in these studies (Table 3) are much lower than the reported PT of 600 µg/L in de-aromatized wine [60]. While Pineau et al. [60] demonstrated that increasing ethyl butanoate concentration by 200 µg/L can enhance fruity perception in red wines, the maximum difference (approximately 100 µg/L) observed within the ranges in the two aforementioned studies dedicated to white wines remains below this value, making it difficult to draw any conclusions regarding its impact on wine aroma.

Regarding ethyl hexanoate, consistent rootstock effects have been reported in white wine by Ziegler et al. [30] and Vilanova et al. [29], and in red wine by Martilla et al. [8]. In Riesling, concentrations ranged from 881 to 1876 µg/L and from 1561 to 1990 µg/L, with a consistent trend across vintages: wine from 140Ru was associated with the highest level and wine from Börner with the lowest [30]. However, this trend was not observed for the other rootstocks studied. In Albariño, lower concentrations have been found than in Riesling, with concentrations ranging from 272 µg/L in wine from 161-49C to 680 µg/L in wine from 110R [29]. Overall, no consistent rootstock trend was observed across the different years and studies for ethyl hexanoate. Similarly in red wine, Martilla et al. [8] found an inconsistent rootstock trend across years for Shiraz. However, in one vintage, where the rootstock effect was significant, wine from 110R was associated with the highest levels of ethyl hexanoate, a finding that shows some similarity to Albariño [29]. However, the absence of quantitative concentration data in some red wine studies makes it difficult to directly evaluate the sensory impact of this compound. Nevertheless, the literature reports a PT in model wine of 440 µg/L for ethyl hexanoate [60], which is well below the concentrations found consistently in Riesling and sometimes in Albariño wines from different rootstock, suggesting a possible direct impact on wine aroma. Moreover, Pineau et al. [60] showed that increasing ethyl hexanoate concentration from 386 to 707 µg/L in dearomatized red wine enhanced fruity perceptions. These concentrations are comparable to the levels observed in Albariño wines, suggesting that rootstock-induced variations in ethyl hexanoate may have an impact on fruitiness.

Regarding ethyl octanoate, consistent rootstock effects were observed in both white and red wine (Table 3). In Riesling, concentrations ranged from 1478 to 3800 µg/L in one vintage and from 3288 to 3903 µg/L in another [30]. A consistent trend across both vintages was identified: wines from 140Ru, 3309C, and Gravesac were associated with the highest levels, while wines from Börner, 110R, and SO4 were linked to the lowest concentrations. In Albariño, a rootstock effect was observed in only one vintage, with overall concentrations lower than those in Riesling (101 to 513 µg/L) [29]. The trends for SO4 and Gravesac were similar to those observed in Riesling, but for 110R and 3309C, they were the opposite. In red wine, Carrasco-Quiroz et al. [21] reported a range of 580–1010 µg/L for ethyl octanoate in Merlot, with the lowest levels observed in wines from 110R and Gravesac, and the highest in wines from 1103P and own-rooted vine. Similarly, Awale et al. [19] found that wine from 1103P (30 and 257 µg/L) were associated with the lowest concentrations, while own-rooted vines (68 and 391 µg/L) were linked to the highest in both vintages. By contrast, Martilla et al. [8] observed different trends across two vintages for ethyl octanoate in Shiraz, with own-rooted vines leading to the highest concentration in one year (similar to Awale et al. in Chambourcin [19] and the lowest in the other. The impact of ethyl octanoate on wine aroma has been reported even at concentrations lower than its PT (960 µg/L, [60]). Increasing the concentration of ethyl octanoate from 358 to 699 µg/L in dearomatized wine enhanced fruity perceptions [60]. However, given the wide variations in concentration ranges observed across the cited studies and the inconsistent rootstock trends across cultivars and vintages, it remains challenging to draw definitive conclusions about the consistent sensory impact of rootstock-induced variations in ethyl octanoate.

Ethyl decanoate consistently shows a rootstock effect in various studies (Table 3). In red wines, Carrasco-Quiroz et al. [21] observed concentrations in Merlot wine ranging from 170 to 260 µg/L (own-rooted–1103P), while a partial effect depending on the vintage was reported in Chambourcin by Awale et al. [19] (14–26 µg/L, 3309C–own-rooted) and in Shiraz wine by Mantilla et al. [8] (1103P–own-rooted). Similarly, in Albariño white wine, Vilanova et al. [29] found a partial effect with concentrations ranging from 182 to 254 µg/L (3309C–196-17Cl). The concentrations of ethyl decanoate found in Merlot and Albariño wines are comparable to those generally reported in young red wines (15–215 µg/L in young red wine), and in some cases they exceed the 200 µg/L PT in model wine solutions [61]. When examining the rootstock trends across different studies, several consistent patterns emerge. In Carrasco-Quiroz et al. [21], own-rooted vines, SO4, Gravesac, and 101-14MGt were associated with the lowest concentrations of ethyl decanoate, while 1103P, 110R and 140Ru exhibited the highest. By contrast, Awale et al. [19] observed that SO4 was linked to the highest concentrations in Chambourcin. This study also found that own-rooted vines were associated with the highest levels, while 1103P and 3309C were linked to the lowest concentrations. Mantilla et al. [8] reported similar trends for the same three rootstocks, reinforcing the observations made by Awale et al. [19]. Finally, in Albariño, the trends mirrored those observed by Carrasco-Quiroz et al. [21] in Merlot for 110R and Gravesac, which were associated with the highest concentrations, while SO4 was linked to the lowest, as observed by Awale et al. [19], and 3309C showed similar trends to those seen by Mantilla et al. [8].

Finally, few results have been published on other compounds such as ethyl dodecanoate, ethyl tetradecenoate, and ethyl hexadecanoate. Romero et al. [22] demonstrated that wine from 1103P was linked to the highest levels of ethyl dodecanoate, while 110R was associated with the lowest (136–399 µg/L). However, these results are based on the cumulative values from the two years of study. In addition, Awale et al. [19] observed a rootstock effect only in one vintage for ethyl tetradecenoate (3.3–4.6 µg/L) and ethyl hexadecanoate (34.2–48.8 µg/L), with own-rooted vines associated with the lowest concentrations and 1103P with the highest. Given the limited and often inconsistent data available for these three compounds, drawing definitive conclusions about the rootstock effects on their concentrations remains challenging.

Regarding AEE, ethyl 2- and ethyl 3-methylbutanoate showed significant effects in Shiraz (Schwarzmann highest; own-rooted lowest; [8]), but no effect was found in Pinot noir [23].

#### 2.4.2. Hydroxylated Ethyl Esters

The effect of rootstocks on HEE and AHEE has been investigated in only two studies, which focused on the Shiraz and Monastrell grape varieties (Table 3). In Monastrell wines, Romero et al. [22] found that ethyl-3-hydroxybutanoate concentrations ranged from 133 to 187 µg/L, with the highest levels observed in wines from 1103P and 110R and the lowest in wines from 41B and 140Ru rootstocks. However, drawing conclusions is challenging since the reported concentrations are cumulative across two years of study. Similarly, using a semi-quantitative approach, Martilla et al. [8] observed a consistent rootstock effect on ethyl 4-hydroxybutanoate in Shiraz wines across two years. Specifically, wine from Schwarzmann was associated with the highest levels, while wines from 1103P and own-rooted vines with the lowest. Thus, overall, it is difficult to draw conclusions about the effect of rootstocks, since the statistical methods used in these studies were not the same.

#### 2.4.3. Higher Alcohol Acetates

After ethyl esters, higher alcohol acetates comprise the most extensively studied family regarding the effect of rootstock. Within this group, the most investigated compounds are 2-phenylethyl acetate and hexyl acetate and 3-methylbutyl acetate (Table 3).

Numerous studies have demonstrated a significant rootstock effect on 2-phenylethyl acetate in both white and red wines (Table 3). In white wines, reported concentrations were higher in Albariño than in Riesling. Specifically, in Albariño the values ranged from 183 to 460 µg/L (420A-110R) and from 230 to 429 µg/L SO4–110R) [29], while in Riesling they varied between 22 and 30 µg/L (140Ru–3309C) and between 18 and 32 µg/L (Börner–140Ru) [30]. Wines from 3309C and 110R consistently contained the highest levels of 2-phenylethyl acetate, while no specific trend was observed for the other rootstocks [30]. Interestingly, Albariño wine from 110R was consistently associated with the highest levels of 2-phenylethyl acetate, similar to the trend observed in Riesling. Furthermore, for both varieties, a rootstock effect was observed across all the studied vintages. In red wines, the rootstock effect was also significant, but the overall concentrations were lower. However, some discrepancies have been noted between studies. Awale et al. [19] reported that wine from own-rooted vines were associated with the lowest concentrations of 2-phenylethyl acetate (23 and 9.8 µg/L), while those from 1103P showed the highest levels (46 and 15 µg/L) in Chambourcin. Conversely, in Shiraz, an opposite trend was observed, with wine from own-rooted vines exhibiting higher levels and 1103P the lowest [8]. However, the concentrations reported in these studies remained well below the PT of 2-phenylethyl acetate in dearomatized wine (6500 µg/L; [62]).

Regarding hexyl acetate, similar results were observed, with a consistent rootstock effect in white wines and some red wines (Table 3). In Riesling, a clear trend was identified, with wines from Börner (67 and 63 µg/L) and SO4 (72 and 75 µg/L) being consistently associated with the lowest concentrations across all vintages, while wine from 3309C (127 and 171 µg/L) were linked to the highest concentrations [30]. In Albariño, concentrations ranged from 16 to 103 µg/L (161-49C–420A) in one year [29]. Additionally, a similar trend was observed in both studies, with wine from SO4 being associated with the lowest concentrations and 3309C with the highest. In Shiraz wines, Martilla et al. [8] reported that wine from own-rooted vines consistently showed the highest levels, while wine from 1103P and Schwarzmann were associated with the lowest. However, a direct comparison with other rootstocks is not possible, as the rootstocks differed across the studies. Regarding the other previously described compounds, the concentrations were found to be significantly below the PT of 670 µg/L in dearomatized wine [62].

A rootstock effect on propyl acetate and butyl acetate has also been highlighted, but only in one study [8].

For 3-methylbutyl acetate, a rootstock effect has been consistently observed in white wines, while in red wines, it appears to have been more variable (Table 3). In Riesling, a specific trend was identified, the wine from Börner being systematically associated with the lowest concentrations (730 and 1018 µg/L). However, the rootstocks linked to the highest concentrations varied between years: in one vintage, Gravesac exhibited the highest levels (2183 µg/L), while in another, 140Ru reached the highest concentration (2368 µg/L) [30]. Similarly, in Albariño, the rootstock effect was observed consistently throughout the study, yet no specific trend emerged, with 3-methylbutyl acetate concentrations ranging from 2687 to 5372 µg/L (196-17Cl–41B) and 868 to 3329 µg/L (161-49C–420A) [29]. The concentrations reported in these two white wine studies generally align with the broader literature on dry white wines (average of 2235 µg/L, *n* = 31; [63]), though Albariño levels are notably higher than Riesling. The 3-methylbutyl acetate concentrations in Riesling are typically below or near the PT of 3900 µg/L for white wine [62]. However, Albariño wines can exceed this white wine PT, suggesting there will be a potential sensory impact on young wines or on the late alcoholic fermentation stage.

In red wines, the impact of rootstock has been found to vary depending on the variety and vintage. In Merlot, wine from 101-14MGt has been linked to the lowest concentrations (760 µg/L), while wines from 140Ru, Gravesac and SO4 have exhibited the highest (1150 µg/L) [21]. In Chambourcin, a rootstock effect has been observed in only one year, with wine from own-rooted vines showing the lowest concentration (340 µg/L) and 3309C the highest (765 µg/L) [19]. In Shiraz, the wine from Schwarzmann has been associated with the lowest level and the own-rooted vines with the highest [8], in contrast to the results found in Chambourcin for this same rootstock [19]. Concentrations of 3-methylbutyl acetate in Chambourcin are similar to young red wines (average 507 µg/L, *n* = 22; [63]), while Merlot levels are higher. Further, 3-methylbutyl acetate concentrations are generally lower than the red wine PT of 2000 µg/L [62].

Additionally, in Riesling, the rootstock effect on 2-methylbutyl acetate was consistently observed across all studied vintages, following a similar trend to 3-methylbutyl acetate. Wine from Börner was systematically associated with the lowest concentrations, 69 and 79 µg/L, while the highest concentrations were observed in wine from 110R in one vintage (93 µg/L) and 140Ru (104 µg/L) in the other [30]. However, these concentrations are significantly lower than the PT of 313 µg/L in dilute alcohol solution [54].

Although the current literature does not allow definitive conclusions to be drawn, variations in concentrations of certain fatty acid ethyl esters, such as ethyl butanoate, ethyl hexanoate, and ethyl octanoate in wines from different rootstocks suggest a potential influence of the rootstock, possibly mediated through perceptual interactions. However, consistent rootstock-related trends remain difficult to establish due to variability between studies, cultivars, vintages, analytical approaches and vinification protocols.

Similarly, higher alcohol acetates are generally present at concentrations below the sensory PT, except for 3-methylbutyl acetate in white wines; moreover, the rootstock effects appear to be more consistent. For other ester families, including alkylated ethyl esters and hydroxylated ethyl esters, the limited data and differing methodologies hinder the identification of clear patterns.

### 2.5. Alcohols

Alcohols are major volatile compounds found in wine, influencing its aromatic profile [64,65]. They are mainly derived from alcoholic fermentation [66] and can be gathered into different categories, including higher alcohols, benzylic alcohols [66], and C6 alcohols [67].

The impact of rootstocks on the production of alcohols in wine has been widely studied in the literature, with a focus on 36 different compounds. These include 19 higher alcohols, five other alcohols, and 12 C6 compounds. However, only compounds whose concentrations or sensory impact are considered relevant were retained for discussion; these are summarized in Table 4.

**Table 4:** Effect of rootstock on concentration range of alcohols in wine.

Aroma Compound	Concentration Range in Wine (µg/L)		Grape Variety Studied		Rootstock Effect		
	White	Red	White	Red	Yes	No	Sometimes
**Higher alcohols**							
Propan-1-ol		94–35,000 [14,22]	Albariño	Monastrell, Shiraz, Merlot, Cabertnet-Sauvignon, Albarín Negro, Verdejo Negro	[14,22]	[16,21,28,29]	[8]
2-Methylpropan-1-ol	167–674 [29]	75,000–96,000 [14]	Albariño	Cabernet-Sauvignon, Monastrell, Merlot, Albarín Negro, Verdejo Negro	[14]	[16,21,22,28]	[29]
3-Methylbutan-1-ol	168,000–363,200 [30]		Riesling	Merlot, Pinot noir	[30]	[21,23]	
**Benzylic alcohols**							
Benzyl alcohol		90–420 [21]	Albariño	Merlot, Shiraz	[21]	[29]	[8]
2-Phenylethanol	3414–27,000 [29,30]	137,360–238,970 [21]	Riesling, Albariño	Merlot, Shiraz, Monastrell, Albarín Negro, Verdejo Negro	[8,21,29,30]	[14,22,28]	
**C6 Compounds**							
Hexan-1-ol	1100–2800 [30]	314–1240 [20,21,22]	Riesling, Albariño	Merlot, Shiraz, Marselan, Monastrell	[8,20,21,22,30]	[29]	
(E)-2-Hexenal		118–210 [20]		Merlot, Marselan	[20]		
(E/Z)-2-Hexen-1-ol		26–41 [20]		Merlot, Marselan, Shiraz	[20]		[8]
(E/Z)-3-Hexen-1-ol	7–50 [29]	0.9–1880 [20,21]	Albariño	Merlot, Marselan, Shiraz	[20,21,29]		[8]

Concentration range: mean concentration recorded for the rootstocks associated with the lowest and highest concentration, only when a statistically significant rootstock effect was observed. Rootstock effect: the categories indicate whether a statistically significant effect of the rootstock was observed in all cases (Yes), in no cases (No), or only in certain cases, varying depending on the vintage (Sometimes).

#### 2.5.1. Higher Alcohols

Higher alcohols include compounds with more than two carbons, mainly produced by yeast through the Ehrlich pathway from amino acids via deamination, decarboxylation, and reduction to alcohols. They can also be formed from α-keto acids derived from sugar metabolism, which enter the Ehrlich pathway [68]. The formation of these compounds is influenced by various factors, including yeast strain, fermentation temperature, must pH, and aeration [69]. Additionally, grape maturity level also affects their final concentrations due to differences in nitrogen levels [70]. Despite their significant presence, the sensory impact of higher alcohols has not been exhaustively studied. These compounds are present in total concentrations ranging from 0.2 to 1.2 g/L in white wines and 0.4 to 1.4 g/L in red wines [71]. Their sensory impact is highly concentration-dependent: at levels below 300 mg/L, they contribute positively to the aromatic complexity of the wine, whereas above 400 mg/L, they can induce harsh and pungent notes, thereby altering perceived quality [71]. More recently, several studies have highlighted their qualitative role in modulating fruity aromas. For example, Cameleyre et al. [64] demonstrated that higher alcohols can alter and mask the perception of fruity aromas in a model wine mixture. Similarly, higher alcohols have been shown to suppress specific aroma notes, such as strawberry/lactic/red fruit, coconut/woody/vanilla, and humidity/TCA, while having no effect on leathery/animal/ink notes in a wine model solution [72].

The effects of rootstock on higher alcohols have been the subject of several studies, though results vary depending on grape variety and vintage. Most research has been conducted on red wines, with limited studies on the effects of rootstocks in white wine (Table 4). The findings indicate that most studies have not found significant differences in higher alcohols. Overall, fermentation conditions are likely the most important factor affecting these compounds, often overshadowing rootstock influence.

The most studied higher alcohols is propan-1-ol, as presented in Table 4. Specifically, in all studies, wine from 110R rootstock has been consistently associated with lower concentrations of propan-1-ol, which has been observed in Monastrell, Shiraz, and Albarín Negro wines. In Monastrell, propan-1-ol concentrations have been found to range from 94 to 226 µg/L over two years, with wine from 140Ru and 110R rootstock being associated with the lowest levels, and wine from 1103P with the highest [22]. These concentrations are markedly lower than average values reported in red wines in the literature, which are typically around 20–30 mg/L [73,74], representing a more than a 100-fold difference.

A similar trend has been observed in Shiraz: wine from 1103P was again associated with the highest propan-1-ol concentrations, while wine from Schwarzmann and 110R exhibited the lowest levels [8]. In Albarín Negro wine, propan-1-ol concentrations ranged from 20 to 35 mg/L over three years, with 110R associated with the lowest levels and 196-17Cl with the highest [14]. These concentrations remain well below the PT of 500 mg/L reported for propan-1-ol in wine solution [66]; meanwhile, values reported by Romero et al. [22] are inconsistent with those commonly found in red wines [73,74], suggesting that sensory differences due to this compound are most likely limited.

Butan-1-ol has been studied by several authors [8,21,22,23,29], but a rootstock effect was highlighted in Shiraz only, with a partial effect being observed in one vintage. In this study, wine from 1103P was associated with the lowest levels, and wine from Schwarzmann with the highest [8].

Rootstock effects on 2-methylpropan-1-ol and 3-methylbutan-1-ol, have primarily been observed in white wines (Table 4). Regarding 2-methylpropan-1-ol, a significant rootstock effect was observed in Albariño wine in one year of the study, with concentrations ranging from 167 in wines from 41B to 674 µg/L in wines from 110R rootstocks [29]. These concentrations are markedly lower than the average values reported for red wines in the literature, which are typically around 35–45 mg/L in red wine, representing a more than 52-fold difference [74]. By contrast, in Albarín Negro, the rootstock effect was reported over a three year period, with concentrations ranging from 75 to 96 mg/L for the 196-17Cl, 101-14MGt, and 3309C rootstocks [14]. These values are consistent with those reported in the literature for red wines [52] and reach twice the PT of 40 mg/L in model wine solution [61], with up to 20 mg/L variation between rootstocks, suggesting a possible impact on wine aroma.

Regarding 3-methylbutan-1-ol (wine PT 300 mg/L; [66]), a rootstock effect was noted only in Riesling over two years. In one year, concentrations ranged from 225 to 363 mg/L in wine from Gravesac and 110R, while in the other year, concentrations ranged from 168 to 225 mg/L in wine from 3309C and Börner. No consistent trend was observed across the years [30]. Using a complex mixture containing about 200 mg/L of butan-1-ol, Cameleyre et al. [64] showed that additions of 3-methylbutan-1-ol at levels of about 80 and 140 mg/L led to aroma changes. Thus, the concentration differences observed here suggest that, in some cases, the observed rootstock effect on 3-methylbutan-1-ol levels could be at the origin of sensory differences.

#### 2.5.2. Benzylic Alcohols

Among the benzylic alcohols, 2-phenylethanol (PT of 14 mg/L in wine model solution; [61]) has been the most extensively studied. Studies on its concentration show inconsistent rootstock effects, with some reporting a consistent rootstock influence and others finding no significant impact (Table 4).

In white wines, a consistent rootstock effect on 2-phenylethanol has been observed across all vintages studied. In Albariño, concentrations ranged from 3.4 to 5.4 mg/L (420A–110R) and from 5.5 to 25 mg/L (RGM–110R) [29]. Similarly, in Riesling, concentrations varied between 10 and 27 mg/L (Gravesac–110R) and 10 to 15 mg/L (SO4–Börner) [30]. Interestingly, 110R was frequently associated with higher levels of 2-phenylethanol in both studies. In red wines, concentrations of 2-phenylethanol were generally higher than in white wines. In Merlot, concentrations ranged from 137 for wine from SO4 to 239 mg/L for 101-14MGt rootstocks [21]. These concentrations are notably higher than those reported for young red wines, which typically range from 40 and 154 mg/L [61]. In Shiraz, an interesting trend was highlighted by Mantilla et al. [8], with wine from 1103P being associated with the lowest levels and Schwarzmann with the highest in both vintages studied.

In white wines, although the observed concentrations of 2-phenylethanol can be higher than the reported PT, their sensory impact might be limited. A study showed that even an increase of up to 80 mg/L in a model wine solution had a negligible impact on perception [72]. This would likely not be the case for Merlot, in which concentrations can reach much higher levels. However, it is important to note that specific winemaking conditions, such as short pre-fermentative maceration and no nitrogen addition, were present in the Merlot study. These protocol differences, rather than the rootstock itself, might explain the observed variations in concentration and their potential sensory impact.

Finally, for benzyl alcohol, a rootstock effect was observed in red wines only (Table 4). Interestingly, in both Merlot and Shiraz, a similar trend was observed for own-rooted vines and 1103P. In both studies, lower concentrations were associated with wine from own-rooted vines (90 µg/L in Merlot), while higher levels were found in 1103P (420 µg/L in Merlot) [8,21].

Based on the studies reviewed here, it can be concluded that while certain rootstocks can significantly influence the concentration of specific higher alcohols, particularly propan-1-ol, the overall impact appears to be often overshadowed by winemaking parameters, such as fermentation conditions and nitrogen availability.

#### 2.5.3. C6 Alcohols

C6 alcohols have long been considered aromatic markers of vegetative/herbaceous character in red wines [75,76,77]. However, recent research has demonstrated that these compounds do not directly contribute to green aromas in red wine [78]. These volatile compounds, which contain six carbon atoms, are derived mainly from the enzymatic oxidation of polyunsaturated fatty acids present in grapes, such as linoleic and α-linolenic acids. These fatty acids are first transformed into C6 aldehydes via the lipoxygenase pathway, then reduced to their corresponding alcohols by alcohol dehydrogenases [79]. The perception threshold of these compounds in hydroalcoholic solution varies significantly, ranging from 400 µg/L for (Z)-3-hexen-1-ol to 8000 µg/L for hexan-1-ol [80], and their abundance depends on pre-fermentative processes [81].

Various C6 compounds have been studied, with hexan-1-ol being the most investigated, and most research has demonstrated a rootstock effect on its concentration (Table 4). In red wine, the rootstock effect on hexan-1-ol has been observed in several studies. In Monastrell, concentrations ranged from 314 to 471 µg/L (140Ru–1103P) over two vintages values [22]. Similarly, in Shiraz, wine from 1103P contained the highest hexan-1-ol levels; however, in another year of the same study, the trend for 1103P was reversed [8]. For Merlot, contrasting trends have been reported. One study found concentrations of between 670 and 1240 µg/L (99R–own-rooted vine) [21], while another found much lower values (53–82 µg/L, own-rooted vine–5BB) [20]. Additionally, the same authors explored the rootstock effect in a single-vintage study and found varying concentrations depending on the rootstock in Marselan wines, ranging from 45 to 65 µg/L (5BB–1103P) [20]. For white wines, conflicting results were reported for hexan-1-ol. In Riesling, a consistent rootstock effect was observed, with a stable trend across vintages, particularly for SO4 and 110R (1100–2800 µg/L for SO4 and 110R and 1300–2800 µg/L for SO4 and Gravesac–110R) [30]. By contrast, no rootstock effect on hexan-1-ol was reported in Albariño [29]. Notably, hexan-1-ol concentrations across most studies are below its PT in water (2500 µg/L; [78]), though concentrations in Riesling can be similar to this value.

Another compound that has been explored in multiple studies is 3-hexen-1-ol, with the rootstock effect reported as either consistent or partial (Table 4). In Merlot, wines from own-rooted vines have generally been associated with lower concentrations, while grafted vines have shown higher values. Specifically, concentrations ranged from 110 to 1880 µg/L (own-rooted vine and 101-14MGt) in one study [21] and 0.9–3.6 µg/L (own rooted vine–5BB) in another [20]. An opposite trend for own-rooted vines has been observed in Marselan, being associated with higher 3-hexen-1-ol concentrations (5.9 µg/L) than in wine from 1103P (4.7 µg/L) [20]. Finally, in Shiraz, Mantilla et al. [8] reported that the rootstock effect was significant in only one vintage, with wine from 1103P being associated with the lowest 3-hexen-1-ol levels and Schwarzmann the highest.

The same authors obtained similar results for 2-hexen-1-ol and 3-hexen-1-ol, with a partial rootstock effect. However, Schwarzmann was consistently associated with the lowest levels of both compounds, and 1103P with the highest [8]. Furthermore, in white wine, a consistent rootstock effect on 3-hexen-1-ol was observed in Albariño, but with no specific trend between rootstocks. Concentrations in wine varied between 11 and 50 µg/L (420A–41B) in one year and 7 to 23 µg/L (161-49C–420A) in another, indicating variability in rootstock influence across vintages [29]. Importantly, 3-hexen-1-ol concentrations can reach 238.1 µg/L, which exceeds its PT of 70 µg/L in water [78].

Finally, no consistent trends have been observed across studies regarding the influence of rootstocks on C6 alcohol concentrations, likely due to fermentation processes being the major contributing factor to these differences. Consequently, evaluating the sensory impact of these compounds in relation to specific rootstock behaviors remains highly complex and often inconclusive.

Although several alcohols, such as propan-1-ol, 2-methylpropan-1-ol, 3-methylbutan-1-ol, 2-phenylethanol, and certain C6 alcohols, can be influenced by rootstock, the magnitude and consistency of these effects vary considerably with cultivar, vintage, and winemaking conditions. In many cases, fermentation parameters and nitrogen management appear to exert a stronger influence than the rootstock itself. Sensory relevance is often limited, because concentrations remain below perception thresholds; however, exceptions do exist, particularly in the cases of 2-methylpropan-1-ol in Albarín Negro, 3-methylbutan-1-ol in Riesling, 2-phenylethanol in Merlot, and 3-hexen-1-ol in certain cultivars, in which levels can exceed their respective thresholds and potentially affect aroma perception.

## 3. Impact of Rootstock on Wine Aroma Perception

Research focusing on the effect of rootstock on wine aroma perception remains limited and mainly addresses red varieties, particularly Shiraz and Cabernet-Sauvignon. Other varieties that have received attention include Monastrell, Albarín Negro, Verdejo Negro, and Alicante Bouschet (Table 1). In these studies, a total of 29 rootstocks were examined, 1103P (*n* = 9), 110R (*n* = 7), and 101-14 MGt (*n* = 6) being the most frequently studied (Table 1). By contrast, research on white wines remains limited, with only two studies conducted on Sauvignon blanc and Vermentino, involving a total of ten rootstocks (Table 1).

Two studies on Cabernet-Sauvignon have investigated the influence of rootstocks on wine using descriptive sensory methods. Miele & Rizzon [17] assessed 15 rootstocks and evaluated olfactory descriptors such as intensity, green bell pepper, fruity, spicy, vegetal, and animal notes, while Somkuwar et al. [18] assessed eight rootstocks and focused on aroma intensity. Both studies involved trained panels of 12 assessors using structured sensory protocols. Despite differences in the number of attributes assessed and scales used, both concluded that rootstock had no noticeable impact on wine aroma.

Among the studies on Shiraz, Walker et al. [25] and Martilla et al. [8] provide the most comprehensive data; their studies were both conducted over two vintages using descriptive analysis and trained panels. Walker et al. [25] evaluated eight olfactory attributes (overall fruit, red fruits, dark fruits, confection, oak/wood, green, drain, and pungent), and observed that wines from 101-14MGt and 1103P consistently received the highest scores for dark and red fruit aromas, while wines from M6262 and Ramsey were generally rated as less fruity. Similarly, Mantilla et al. [8], using a broader set of aroma descriptors (fresh red berry, fresh dark berry, floral, confectionary, green, dry fruit, savory spice, sweet spice, and alcohol), reported that wines from Schwarzmann and 1103P often showed the highest intensities for dark berry aromas, while wines from own-rooted vines and 110R recorded lower intensities for the same descriptor. Despite the differences in number of descriptors and in scoring systems, both studies revealed consistent trends, notably the positive contribution of 1103P to fruit-related descriptors. These patterns are partly supported by de Oliveira et al. [27], who compared 1103P and IAC 313 over two harvest seasons using quantitative descriptive analysis. Wines from IAC 313 obtained highest score for fruity and spicy notes, while those from 1103P displayed more floral and herbaceous notes.

Capone et al. [24] used free-choice profiling with eight trained judges and found that wines from Dog Ridge and M6262 were repeatedly described as having stronger green/vegetal aromas, echoing the lower fruit expression associated with M6262 observed by Walker et al. [25]. Although informal, this evaluation complements formal analyses by reinforcing the role of some rootstocks in modulating perceived ripeness and green character. In their evaluation of two rootstocks (5C and Gravesac), Heller-Fuenzalida et al. [26] reported significant differences in meaty aroma, 5C being associated with the highest score. While limited in scope, this study confirmed that rootstock can influence key aromatic descriptors.

In summary, in Shiraz wines, 1103P was the most frequently studied rootstock and it is generally associated with greater fruit expression. By contrast, M6262 tends to produce wines with lower intensity and greener profiles.

As well as Shiraz, several studies have explored the effect of rootstock on other red grape varieties. Romero et al. [22] assessed the interaction between five rootstocks and two irrigation regimes across three vintages of Monastrell, using a structured difference testing protocol with a small panel of five trained tasters. Significant differences were observed for olfactory attributes depending on the rootstock. Wines from 1103P and 41B were consistently associated with the highest olfactory scores, with 1103P rated best for olfactory quality. By contrast, wines from 110R also showed a lower score for olfactory quality. The total score, integrating all phases, confirmed that wine from 41B and 1103P were evaluated as being the best, while wine from 140Ru was evaluated as being the worst [22]. These results, based on three-year averages, underscore a strong and consistent rootstock effect in Monastrell wines, with 1103P again associated with favorable olfactory characteristics, in line with observations made for Shiraz.

Studies by Loureiro et al. [14,28] investigated the effect of rootstock on the sensory characteristics of Albarín Negro (five different rootstocks, [14]) and Verdejo Negro (three rootstock, [28]) wines, also across three vintages and using the OIV scoring sheet. In both studies, the wines were evaluated for olfactory attributes. Although no significant differences were observed, wine from 196-17Cl obtained slightly higher overall nose quality scores in the Albarín Negro study, while wine from 101-14MGt performed marginally better for nose parameters in the Verdejo Negro [14,28]. However, due to the lack of information on the tasting panel in Loureiro et al. [14] and the limited number of assessors (*n* = 6) in Loureiro et al. [28], it is difficult to assess the robustness of these results.

In addition to red varieties, two recent studies have reported the impact of rootstock on white wine sensory characteristics, focusing on Sauvignon blanc, and Vermentino (Table 1). In Sauvignon blanc study, seven rootstocks were evaluated over one vintage using a hedonic test with six assessors. Aroma was the only olfactory attribute assessed, with wines from 110R receiving the highest scores and those from 1103P the lowest [31].

The Vermentino study by Nardello et al. [32] applied a more structured approach, involving ten trained judges over three vintages. Wines were evaluated using a 0–10 intensity scale for olfactory attributes, including floral, spice, fruity, defects, and herbaceous notes. Results showed more herbaceous notes and defects in 2019, especially in wines from Harmony and VR 043-43. These olfactory differences, particularly in terms of defects, could also potentially be attributed to variations in the vinification process rather than to the rootstock itself. However, no consistent sensory pattern was observed across vintages for individual rootstocks, suggesting that vintage effects may have outweighed rootstock influence [32]. Moreover, no aroma compound analyses or correlations between sensory and analytical data have been reported, making it difficult to draw sound conclusions on the link between wine composition and perception.

Overall, the direct sensory impact of rootstocks on wine aroma is complex and highly dependent on grape variety and experimental methodology. While studies on Cabernet-Sauvignon generally show no significant differences linked to rootstock, Shiraz and Monastrell exhibit clear and often consistent rootstock-driven aroma effects, notably the enhancement of fruitiness associated with 1103P. Other commonly studied rootstocks, such as 110R and 101-14MGt, show more variable effects. Additionally, rootstocks like M6262 and Dog Ridge tend to be associated with vegetal attributes. However, the range of varieties and rootstocks that have been extensively investigated is limited, and methodological challenges exist in studies on other red varieties and white wines; this thus underscores the need for more robust and comprehensive investigations if definitive conclusions are to be drawn across diverse wine types.

## 4. Conclusions and Perspectives

### 4.1. Key Findings

Available research on the effects of rootstocks on wine aroma compounds and perception was synthesized in this review. The existing literature on the subject is fragmented, with only a small fraction of the vast rootstock diversity used in viticulture having been explored. A total of 33 rootstocks have been studied for their influence on volatile compounds, and 29 for sensory evaluation. However, most rootstocks have only been investigated in a single study, leaving their genetic potential and behavior under different environmental and viticultural conditions largely unexamined. By contrast, a few rootstocks, such as 1103P, 110R, 3309C, and SO4, have been the most frequently evaluated across multiple grape varieties and environmental conditions.

While some rootstocks have shown more consistent effects on aroma compounds (for instance, 3309C often associated with higher levels of β-damascenone and hexyl acetate), consistent trends remain scarce. Indeed, the influence of rootstocks on aroma compound concentrations appears to be highly context-dependent, and very few effects are consistently observed across different experiments. In most cases, the same rootstock does not systematically lead to similar changes in the wine’s aroma compounds. Furthermore, a significant gap exists in the types of aroma families studied. Research has predominantly focused on esters (particularly fatty acid ethyl esters and higher alcohol acetates), as well as on higher alcohols, and monoterpenic compounds. Conversely, other important aroma families, such as methoxypyrazines, C13-norisoprenoids, hydroxylated ethyl esters, and C6 compounds, have received comparatively limited attention. Notably, some families, such as volatile thiols, lactones, and furanones, have not been explored at all in this context.

Beyond the limited scope of aroma families studied, the overall methodological disparities across studies represent a major challenge, creating substantial confounding factors that hinder the isolation of rootstock-specific effects. The variability in winemaking protocols, particularly in nitrogen management, fermentation conditions, and aging practices, further complicates the isolation of rootstock-specific effects. For instance, varying approaches to malolactic fermentation management, such as spontaneous malolactic fermentation [16], lactic bacteria co-inoculation during alcoholic fermentation [8], or post-alcoholic fermentation inoculation [14], can significantly influence wine chemical composition.

Similarly, other practices like short pre-fermentative maceration [21] or variations in nitrogen management before alcoholic fermentation, ranging from specific additions [8,30] to a frequent lack of detailed reporting on any such practice [16,21], can introduce significant bias. These non-standardized practices can then alter the wine’s aroma compounds and overall aromatic expression.

Similarly, the timing of sample analysis further complicates interpretation, given that wine aroma composition evolves significantly in different storage or aging conditions. For example, in some studies, wines were aged for the same amount of time (3 months), but in different temperature conditions (±5 °C, [20]; 15 °C, [8]). Furthermore, reporting is inconsistent, with some studies considering together samples from different vintages [16] while others do not specify precisely at which winemaking stage the wines were analyzed [21].

In addition, sensory analyses remain highly variable in terms of methodology, often involving small or untrained panels, and they lack sufficient replication or standardization. Only a few rootstock × cultivar combinations have been explored over multiple vintages, and only a very limited number of studies (exclusively on red cultivars) have simultaneously assessed both aroma compounds and perception.

Altogether, these limitations underline the necessity of interpreting the current literature with caution. While certain patterns emerge (e.g., impacts of 3309C), the same rootstocks do not consistently produce the same effects across different studies, grape varieties, or conditions.

### 4.2. Future Directions

To make advances in this field, future research should focus on more standardized, replicated, and long-term studies that combine robust analytical chemistry with standardized sensory analysis. Such studies should broaden the diversity of rootstock and scion genotypes and better disentangle the highly context-dependent interactions among rootstock, scion, and environment that ultimately drive grape and wine composition. In addition, the genetic basis underlying rootstock influence on grape and wine metabolites remains little explored, highlighting the need to couple classical viticultural trials with molecular and biochemical approaches.

Although rootstock choice in viticulture is still largely driven by agronomic criteria (vigor, yield, tolerance to abiotic and biotic stresses), its enological consequences, particularly for wine aroma compounds and sensory perception, are rarely considered. The results of studies by Ziegler et al. [30] and Vilanova et al. [29] indicate that some aroma compound contents could be related to yield. The R^2^ values we calculated from data reported in these two studies showed a significant relationship between linalool level and yield in Riesling (R^2^ = 0.69; [30]) but not in Albariño [29]; this suggests there was an interaction with the scion varieties studied. Moreover, a significant relationship between yield and TDN was observed in one of the studies (R^2^ = 0.41; [30]), but no relationship was found between yield and its contents for other aroma compounds, such as β-damascenone, based on data from Ziegler et al. [30] and Vilanova et al. [29]. Therefore, these results highlight the potential influence of vineyard agronomic parameters on wine aroma compounds. In particular, yield (as presented above) and vigor (as indicated by the Pearson coefficients of 0.47 (vigor vs. TDN) and 0.61 (vigor vs. vitispiranes) reported by Ziegler et al. [30], should also be considered, along with envirotyping (i.e., characterizing the environment in terms of water and nitrogen status). Bridging this gap requires integrated approaches that accounts for agronomic variables across the vine growth cycle, such as phenological development and precocity, vegetative growth and canopy size, yield components, water and mineral status, together with the modulating effects of vineyard and winemaking practices. Such interdisciplinary frameworks are essential to understand how rootstocks influence both vine performance and wine sensory quality.

## Data Availability

No new data were created or analyzed in this study.
